# Barriers and Facilitators Perceived by Spanish Experts Concerning Nursing Research: A Delphi Study

**DOI:** 10.3390/ijerph17093224

**Published:** 2020-05-06

**Authors:** Alberto González-García, Ana Díez-Fernández, Noelia Martín-Espinosa, Diana P. Pozuelo-Carrascosa, Rubén Mirón-González, Montserrat Solera-Martínez

**Affiliations:** 1 Centro de Estudios Sociosanitarios, Facultad de Enfermería de Cuenca, Universidad de Castilla-La Mancha, 16071 Cuenca, Spain; alberto.gonzalez@uclm.es (A.G.-G.); montserrat.solera@uclm.es (M.S.-M.); 2 Grupo de Investigación Multidisciplinar en Cuidados (IMCU), Facultad de Fisioterapia y Enfermería de Toledo, Universidad de Castilla-La Mancha, 45071 Toledo, Spain; noelia.martin@uclm.es (N.M.-E.); DianaP.Pozuelo@uclm.es (D.P.P.-C.); 3 Facultad de Medicina y Ciencias de la Salud, Universidad de Alcalá, Alcalá de Henares, 28805 Madrid, Spain; ruben.miron@uah.es

**Keywords:** nurses, nursing research, Delphi method, consensus, Spain

## Abstract

The identification of research priorities in line with current health needs and nursing competencies is a priority. Nevertheless, barriers and facilitators perceived by nurses to performing nursing research have scarcely been investigated. The main aim of this study was to explore the situation in nursing research in Spain, as perceived by Spanish experts. A Delphi study technique in two phases was applied using an online survey tool. A panel of 20 nursing experts in nursing, teaching and management positions participated. The strengths highlighted were the possibility of reaching the PhD level, the possibility of receiving continuous training in research methodology, and access to scientific knowledge through the Internet. The weaknesses identified were the lack of Spanish nursing journals in which to publish the research results, the lack of funding in nursing care research, and the lack of connection between the healthcare institutions and the university. According to the experts, elements that could enhance leadership in research are the creation of nursing research units in hospitals, the economic recognition of nurses with PhDs, and considering research work as part of their daily tasks in clinical settings. The idea of being subordinated to physicians still remains in nurses’ ways of thinking.

## 1. Introduction

Over the past two decades, several factors have led to an increase in demand for qualified nursing care: changes in providing health care, economic cutbacks in healthcare organization, and technological developments. Bäck-Pettersson et al. [1] have suggested that nursing research should be geared towards the study of principles for effective and efficient nursing practice and factors affecting perceptions of health and well-being among individuals, families, communities and healthcare services.

The changes which nursing has undergone in Spain in the last two decades, both at an academic and professional level and as to the evolution of the health system, have drawn a favorable framework for the progressive incorporation of nursing professionals in the activities of scientific knowledge generation and communication [2]. Thus, the identification of research priorities in line with current health needs and nursing competencies is a priority in itself.

The Delphi method has been shown to be a widely used and flexible method that is particularly useful in achieving consensus in a given area of uncertainty or lack of empirical evidence [3]. It seeks to gain the most reliable consensus of a group of experts by answering a series of sequential questionnaires or “rounds” intermingled by controlled feedback [4,5]. The Delphi technique has been used in a wide variety of nursing research, such as for determining the priorities of nursing administration research [6] and for the delivery of health care to children [7,8,9]. With regards to the academic field, the quality of nursing doctoral education and strategic directions for improving quality have been pointed out [10], as well as the identification of characteristics and essential elements of lifelong learning [11,12].

To our knowledge, only one study has tried to identify the barriers and facilitators perceived by nurses to the use of nursing research [13], focused on the Chinese context. The main difficulties identified were lack of authority, lack of time, language difficulties, lack of financial resources and lack of legal protection. The elements in favor were an improvement in management support, the promotion of education to increase the knowledge base and the moderate increase in the time available for implementation. The health center, academic training, and knowledge of the theoretical basis of evidence-based nursing were the factors that influenced these barriers and facilitators’ perceptions [13]. Nevertheless, interest in Spain has been focused on establishing research priorities in healthcare [14,15], but not in identifying the weaknesses, threats, strengths and opportunities to research in nursing. In both cases, a list of closed priorities that had been previously agreed upon in a working group was used, but professionals in the field of clinical practice were not included in the group of experts.

It has been recommended to periodically reevaluate the academic programs of the nursing teaching centers to ensure that their approach is adapted to the current challenges, thus improving productivity [16]. In a context in which research excellence is a priority [17], and taking into account that access to external funding sources for the development of research projects requires a high competitive level, it seems logical to examine the current situation to know if it is necessary to make changes. In this sense, the Delphi method itself can provide elements for planning future scenarios [18].

Nurses play a pivotal role in the delivery of effective health care [8]. Considering that nurses and midwives comprise almost 40% of the healthcare workforce, the care that they deliver has a significant impact upon patient outcomes [19]. However, concerns about whether nurses and midwives use the best available evidence to guide their clinical practice have been noticed [20,21]. A lack of research use by nurses and midwives has potentially damaging consequences, with up to 30%–40% of patients not receiving appropriate care [22].

For this reason, the main aim of this study was to explore the situation in nursing research in Spain, as perceived by Spanish experts. Secondary aims were to create a consensus on barriers and facilitators to delimit the characteristics of nursing research in Spain at present, and, finally, to identify the difficulties that clinical-care nursing professionals encounter in carrying out this task.

## 2. Methods

### 2.1. The Delphi Method

This study was conducted using the Delphi method. This method of study was developed in the 1950s by the RAND Corporation for military use [23]. The Delphi approach is a structured process that utilizes a series of questionnaires or rounds to gather and to provide information without the need for face-to-face meetings. The process continues until group consensus is reached. It is growing in popularity, especially for nurse researchers and for health research in general. It provides a relatively rapid and efficient way to obtain agreement from a wide variety of key informants by presenting a series of questionnaires for ranking. Maintenance of participation levels at 50% to 80% is crucial in reaching the consensus [24].

Google forms™ was used as the vehicle for distribution of the questionnaires. Participants were provided the link to the questionnaires through email correspondence. The study had two rounds.

In addition to sociodemographic and educational characteristics of the panel members, which are regarded as crucial to enable assessment of their credibility, round one was used to generate ideas [25]. Participants were asked two open-ended questions, thus allowing panel members freedom in their responses. These questions were (a) “Comment and describe what are the main strengths, opportunities and elements in favor of nursing in order to carry out your research activity. In other words, what facilitates and/or would facilitate our research work?”; and (b) “Comment and describe what are the main weaknesses, threats and difficulties and elements against nursing in order to carry out your research activity. In other words, what obstructs or would hinder our research work?”.

Two researchers read the responses from the open-ended questions in the round one independently, searching for relationships and patterns and classifying them into clusters of similar ideas. The most commonly occurring attributes and concepts were identified and grouped into similar ideas. To validate the concepts that occurred, the individual notes were compared, and a third researcher evaluated the areas that deferred. Two researchers developed then the concepts that most commonly occurred into statements that retained the panelists’ collective conceptual meaning. Statements were finally classified into four categories, following the structure of a strengths, weaknesses, opportunities and threats (SWOT) matrix. The number of items generated was 80. After that, two different researchers classified every item into seven main categories created ad hoc: nursing environment, academic level achieved, health administration, support for research from health administration, PhD issues, academic background, and nursing profession.

In round two of the study, participants were asked to rate their agreement with a Likert scale with numerical values attached to the scale range: zero (0) indicated strongly disagree and ten (10) indicated strongly agree. Data were collected between November 2016 and March 2017.

### 2.2. Sample

Purposive sampling stratified by clusters was used for creating the panel of experts. An expert has been defined as one of a group of informed individuals and specialists in their field or someone who has knowledge about a specific subject [25]. Purposive sampling has been widely supported as an appropriate method of sample selection, especially in qualitative research [26]. The most important requisite of purposeful selection is the identification of experts in disciplines or domains directly and indirectly represented in the research instrument or topic under discussion. In addition to this, purposive sampling within maximum variation sampling is one way to obtain representativeness and rich data by including a wide range of extremes [27]. Maximum variation or heterogeneity sampling is described as a special kind of purposive sampling, which may be used to identify experts or cases (in qualitative research) to provide rich information [27]. This sampling method aims to identify themes or patterns that run through a range of variations.

Taking everything into account, the panel of experts consisted of four clusters: (1) nurses actively working at hospitals or primary health-care centers; (2) nurses working as a lecturer or full-time professor at the university; (3) nurses in a management position at a hospital (managing directors or directors of nursing); and (4) nurses in a management position at the university (dean, vice dean or head of the nursing department). These clusters include the professional fields of a nursing professional in Spain.

Regardless of the group to which they belonged, the research team considered it of great importance that the panelists show active research activity, given the topic of the proposed study. For this reason, active research activity was verified prior to its inclusion in the panel of experts in the form of a minimum of five papers indexed in Web of Science (WOS). There is little agreement about the size of the expert panel. Sample size and heterogeneity depends upon the purpose of the project, design selected and period for data collection. For the conventional Delphi, a heterogeneous sample is used to ensure that the entire spectrum of opinion is determined. Moreover, anonymity provides an equal chance for each panel member to present and react to ideas, unbiased by the identities of other participants. Reactions are given independently, so each opinion carries the same weight and is given equal importance in the analysis [25].

A reference group of twenty nurses representing all the clusters mentioned above was regarded as a convenient group of informed individuals and specialists with the requisite expert knowledge concerning nursing research, thus qualifying them as panel members. The principal investigator emailed them individually, explaining the characteristics of the study and proposing them to be part of the panel of experts. All of them responded positively to the invitation.

### 2.3. Validity

Content validity was enhanced in some respects. Firstly, open-ended questions from round one and statements from round two were pretested with a representative sample of nurses to ensure that the concepts included in the study were clear. Secondly, the purposive study sample was comprised of a panel of experts who actively participate in nursing profession [28], so that they are representative of the area of knowledge [29]. Thirdly, successive rounds of the questionnaire increase the validity. Finally, the validity of results will be ultimately affected by the response rates [25]. Nevertheless, the findings should be regarded as expert opinions rather than indisputable data, and the validity and credibility of the research depends on the accuracy of conducting and reporting in the study. It is also important to be aware that the results only represent one moment in time [3,24].

### 2.4. Ethical Considerations

The Ethics Committee of the “Virgen de la Luz” Hospital approved the protocol (registration number 2016/PI0116). Each participant was assigned a code to protect his or her data and affiliation. Informed written consent was required by email. Information was provided on the purpose, risks, benefits and social implications of participation. The right to refuse participation, to decline to answer questions posed or to withdraw at any stage of the process without any penalty or consequence was assured prior to eliciting participation. The ethical principles of the Declaration of Helsinki and the Oviedo Convention of Human Rights and Biomedicine were followed.

### 2.5. Analysis and Consensus

Rated round two responses were recoded into three levels of agreement: 0–4 (low consensus), 5–7 (medium consensus), and 8–10 (high consensus). Only statements classified as high consensus were analyzed. The percentage of high consensus, mean values and standard deviations were calculated. The high consensus statements, with a percentage of ≥ 65%, a mean value of > 8.0 points and a standard deviation of < 2.5 were finally considered as the final conclusion of the Delphi method. Data analysis was performed using the computer package IBM SPSS Statistics version 24 (SPSS, Inc., Chicago, IL, USA).

## 3. Results

As mentioned above, twenty introductory letters were sent (five from each cluster). Of these, eighteen respondents fully completed both rounds (90%). Demographic and educational characteristics of the panel members who responded to all rounds are presented in Table 1. Ten were female and their ages ranged from 33 to 61 years old (mean = 46.61, SD = 9.84). Experts came from every area of expertise equally: registered nurses (*n* = 2), management nurse positions (*n* = 2), lecturers or professors at the university (*n* = 2) and lecturers or professors in management positions (*n* = 2). Almost all of the participants had reached the PhD level (75%).

High-consensus statements that reached the inclusion criteria are presented in Table 2 following a double classification: on one hand, the structure of a SWOT matrix; on the other hand, the main categories created ad hoc and the items. The items comprise a broad spectrum of nursing research topics, from the nursing environment and profession to support from healthcare providers necessary to develop research in nursing.

The strengths highlighted by the experts revolve around three dimensions or categories: academic level achieved, health administration and support from health administration. The most important ideas were the possibility of reaching the PhD level, the possibility of receiving continuous training in research methodology, and access to scientific knowledge through the Internet. These strengths could be reinforced with the following opportunities: the privileged position of nursing to perform clinical research in all areas of health and care levels, the possibility of economic recognition with the PhD level, the creation of specific clinical research units in nursing in the health services, and giving visibility to nurses as researchers.

The weaknesses identified are related to nursing environment, support for research from health administration and the nursing profession: the lack of Spanish nursing journals in which to publish the research results, the lack of funding in nursing care research, and the lack of connection between healthcare institutions and the university. Threats are also related to health administration and the nursing profession. Firstly, “Care research is considered an extra task that must be performed outside the workday” (Participant 9983H). Secondly, the lack of a research mindset among nursing professionals is explained as “There is no culture of research within the profession” (Participant 9990Q). Thirdly, panelists also point out that “The colleagues themselves make it difficult for professionals with research concerns” (Participants 9990Q, 9989P, 9999Z, 9986L).

Finally, a threat related to the historical identification of nursing as a delegate and subordinate profession to physicians with little leadership capacity is detailed as follows: “Become aware that nursing has its own field of knowledge and that it is not subordinate to other sciences such as medicine” (Participant 9997X), “It is not recognized that the nurse can investigate, and being considered as a profession by organizations” (participant 9998Y), and “The persistence of a delegated, subordinate self-image, along with the low ambition of professionals” (9993T).

The rest of the statements that did not reach the minimum consensus required are included in the Supplementary Table S1, as they can help to provide a full picture of the nursing situation in Spain.

## 4. Discussion

The aim of this study was to explore the situation of nursing research in Spain perceived by Spanish experts. To our knowledge, this study is the first to highlight the barriers and facilitators perceived by nurses concerning nursing research in the Spanish context. When it comes to reaching consensus, sharing knowledge, being able to make reflective statements without being personally confronted, and obtaining responses from colleagues in the panel are described as some of the advantages of the Delphi method. Previous studies have pointed out that the panel members affect each other and may change their point of view during the rounds [24]. Moreover, the number of rounds is variable, and when this number is increased, it becomes more difficult to achieve high participation levels [30]. Therefore, two rounds were performed in our study in order not to bias the panelists’ opinions during the process.

When analyzing the international context, some common areas appeared as difficulties for nursing research: lack of time during the workday, language difficulties, and lack of financial resources [13]. Having access to almost all areas and health care levels offers a wide range of possibilities in nursing research, which was previously stated in other contexts [1]. Our study confirmed, as in the Swedish context, that an element in favor of nursing research has been the development of doctoral education programs within nursing science, which was made possible by the increase in the number of registered nurses that have the PhD degree. Certainly, nursing research has increased in both quantity and complexity, according to a recent scoping review that identified four global research priorities: nursing theory development, methodology of nursing research, expertise in advanced nursing and professional nursing practice [17].

While, in a study involving seven countries, it was noted as a concern that research in nursing science may be compromised due to the primary supervision of nursing doctoral students by non-nurse supervisors [10], in our context it does not appear as a threat with a sufficient degree of consensus, although this phenomenon does exist. Nevertheless, it has been recently pointed out that nursing as a discipline is rather young and the theoretical development of nursing knowledge is even more recent [17]. The main difficulty in this field is the lack of connection between the healthcare institutions and the university. Creating nursing research units in healthcare institutions and achieving economic recognition of nurses with the PhD level are considered imperative to increase nursing research.

Previous studies carried out in Spain indicated as main barriers the lack of time during the workday to implement new ideas, that nurses do not perceive as relevant the results of the research for its application in clinical practice and the lack of collaboration of physicians for the implementation of nursing research [31]. Even though they do not coincide exactly with the items that have been proposed in our study, conceptual similarities are found, so that our panelists point out that there is no research mindset among nursing professionals. The lack of collaboration with the medical group was also indicated in round two of our study, but it did not reach a sufficient degree of consensus to be included in the final items. Finally, while, in the first study in Spain, a lack of training in research methodology was pointed out as a weakness [14], our study shows that this difficulty seems to have been overcome because panelists identified it as a strength. In this regard, North American nurses in management positions have recently assured, on the one hand, that they are not prepared with the essential skills to succeed so that training and education are essential. On the other hand, they pointed out that most healthcare institutions have prioritized workflow and productivity over research, which does not favor nursing research [32]. These aspects have been stated in our study.

Our study shows that there is still a self-image as a delegate, subordinate profession with little leadership capacity. This situation is specific to the Spanish context, but it has also been confirmed in other contexts [13,28]. This situation is explained by the historical trajectory of the nursing profession in Spain, given that the profession was conceived as an auxiliary of physicians [33]. Until the transformation from polytechnics to university colleges since 1977, nursing did not become an autonomous profession [34]. Certain factors have previously been stated to enhance nurses’ leadership skills: gaining experience in policy development, having role models and incorporating leadership skills in bachelors’ curricula [28]. Our study shows that academic positions to represent nursing voices are considered a strength in our country, but nothing is mentioned about achieving management or health policy positions.

### Limitations

Our study has several limitations that should be acknowledged. First, this method is not a replacement for rigorous scientific reviews of published reports or for original research. Second, the sample size was small, and this may be considered as a limitation in this study. Nonetheless, it has been stated that a sample of 20–50 experts is recommended for surveys of expert opinions [35]. This type of sampling is particularly useful if the target population from which experts may be drawn is not clearly defined, or if there is great variation in the domain or phenomenon to be studied [36]. In any case, the level of agreement must be set by the researcher prior to implementation; in our case, only high-level consensus and a percentage agreement of > 65% were analyzed. Third, not exploring disagreement or marginalizing dissenting voices may also generate artificial consensus [36]. Fourth, the existence of consensus from a Delphi process does not mean that the correct answer has been found [25], but that a collegiate response has been obtained among the main representatives of the area of knowledge [1], and this response represents an opinion in the instant object of the study; therefore, it may vary over time [24]. Fifth, the sample was selected on purpose, as per the researchers’ knowledge of the contribution that the expert panelists could make to the study. This may have resulted in some relevant nurse leaders being excluded.

## 5. Conclusions

Our study allows us to conclude that the main strengths for nursing research in Spain highlighted by experts were the access and development of PhD programs, as well as the privileged position of nursing in all clinical settings. Elements that could enhance the achievement of leadership in research according to the experts were the creation of nursing research units in hospitals, the economic recognition of nurses with the PhD level, and considering research work as part of the daily tasks of the clinical nurse. Despite the elapsed time, the ideas of being subordinated to physicians and having little leadership capacity apparently remain in nurses’ ways of thinking. Experts pointed out that it is necessary to work on the connection between the universities and the healthcare institutions, as well as on raising awareness among professionals in the clinical field about the need for teamwork to achieve leadership in care research. Given that the findings of a Delphi group represent expert opinion rather than indisputable fact, further inquiry to validate the findings and to develop an international panel of experts from different contexts may be important.

## Figures and Tables

**Table 1:** Characteristics of the study sample (panelists).

Characteristics	Male (*n* = 8)	Female (*n* = 10)	*p* Value
**Age (mean ± SD)**	**41.63 ± 7.58**	**50.60 ± 9.92**	**0.051**
**Degree, n (%)**			
Registered nurse (prior to university level)	0 (0.0 %)	1 (100.0 %)	0.830
Registered nurse (prior to EHEA)	6 (45.5 %)	7 (54.5 %)	
Registered nurse (EHEA, bachelor’s degree)	2 (50.0 %)	2 (50.0 %)	
**Total**	**8 (44.4 %)**	**10 (55.6 %)**	
**Academic level,** **n (%)**			
Bachelor’s degree (RN)	1 (100.0 %)	0 (0.0 %)	0.498
Master’s degree (MSc)	1 (50.0 %)	1 (50.0 %)	
Doctoral level (PhD)	6 (40.0 %)	9 (60.0 %)	
**Total**	**8 (44.4 %)**	**10 (55.6 %)**	
**Work place, n (%)**			
Registered nurse	2 (50.0 %)	2 (50.0 %)	0.930
Management nurse position	2 (50.0 %)	2 (50.0 %)	
Lecturer/Professor at the University	2 (50.0 %)	2 (50.0 %)	
Lecturer/Professor at the University, management position	2 (33.3 %)	4 (66.7 %)	
**Total**	**8 (44.4 %)**	**10 (55.6 %)**	

Abbreviations: SD = standard deviation; EHEA = European higher education area; RN = registered nurse; MSc = Master of Science; PhD = doctorate.

**Table 2:** High-consensus statements after round two.

Strengths	Mean (SD)	% of Agreement
*** Academic level achieved***		
To have achieved direct access to PhD programs	8.78 (1.11)	66.7
To reach academic positions with sufficient power to sustain and represent the voice of nursing	8.39 (1.75)	66.7
*** Health administration***		
Training and permanent updating in research methodology	8.89 (1.41)	77.8
*** Support from health administration***		
Access to scientific knowledge through the Internet	8.67 (1.97)	66.7
**Opportunities**		
*** Nursing environment***		
There is a wide range of possibilities in clinical research	9.06 (1.06)	77.8
Nursing professionals play an important role in almost all areas and health care levels	8.56 (1.38)	66.7
*** Health administration***		
Economic recognition of nurses with PhD level	8.67 (2.35)	77.8
To create nursing research units in hospitals supported by health management	8.78 (2.21)	72.2
*** Support from health administration***		
Give visibility to nurses as researchers	8.78 (2.05)	77.8
**Weaknesses**		
*** Nursing environment***		
There are few journals in the area of nursing in Spain, currently any Spanish nursing journal has JCR impact	8.67 (2.14)	66.7
*** Support for research from health administration***		
Nursing care research funding is not a priority policy	8.72 (1.87)	77.8
*** Nursing profession***		
Lack of connection between healthcare institutions and the university	8.61 (1.20)	66.7
**Threats**		
*** Health administration***		
Care research is considered an extra task that must be performed outside the workday	8.83 (2.30)	83.3
*** Nursing profession***		
There is no research mindset among nursing professionals, or lack of motivation	8.44 (1.91)	66.7
The colleagues themselves make it difficult for professionals with research interests	8.94 (1.11)	66.7
Persistence of nursing self-image as a delegate, subordinate profession with little leadership capacity	8.83 (0.85)	66.7

Abbreviations: SD = standard deviation; PhD = doctorate; JCR = journal citation reports.

## Supplementary Materials

The following are available online at https://www.mdpi.com/1660-4601/17/9/3224/s1, Table S1: Rest of the statements after round two (Low and medium-consensus statements).
