# Supplementary material for: Barriers and Facilitators Perceived by Spanish Experts Concerning Nursing Research: A Delphi Study
Source: Int J Environ Res Public Health. 2020 May 6;17(9):3224. doi: 10.3390/ijerph17093224 (PMC7246773; doi:10.3390/ijerph17093224)
Supplement: Supplementary file 1 [file ijerph-17-03224-s001.pdf]

Supplementary Table S1. Rest of the statements after round two (Low and medium-consensus statements).

**Strengths**	**Mean (± SD)**	**% of Agreement**
Nursing environment		
Positive attitude towards nursing care	8.61 (1.15)	61.1
Being always close to patients	8.33 (1.50)	44.4
Nursing possess the leadership in health education	8.11 (1.57)	44.4
Autonomy at work	7.56 (1.79)	27.8
The eminently practical character of our profession	7.44 (1.79)	22.2
The rapid applicability of nursing research	7.28 (1.84)	22.2
Academic level achieved		
Development of nursing specialties, which facilitates training associated with research and evidence-based nursing practice	8.06 (1.35)	50.0
The emergence of Advance Practice Nursing	7.67 (1.88)	44.4
The existence of master and PhD programs	7.89 (1.88)	38.9
Wide range of postgraduate studies	7.83 (1.30)	38.9
Support for research from Health Administration		
Easy connection via internet with other professionals with the same research interests	7.72 (1.93)	33.3
**Opportunities**		
Nursing environment		
Possibility of being part of multicentric and multidisciplinary research networks	8.72 (1.32)	61.1
Create research groups linked to nursing	8.00 (2.22)	55.6
Nursing has access to population groups potentially included in the health care research field	8.61 (0.85)	50.0
To work in an interdisciplinary team	8.00 (1.90)	44.4
Most of the nursing care areas has not been evaluated yet, so it is an innovative field of action	7.39 (1.50)	33.3
Become aware that nursing discipline has its own field of knowledge and is not subordinate to other health sciences	7.33 (2.47)	27.8
Academic level achieved		
To speak foreign languages. especially English language	8.44 (1.46)	55.6
Health administration		
Financial support for specific training	7.94 (2.75)	66.7
Financial support to attend research events	7.89 (2.74)	61.1
To achieve free days or reduction of hours within the working day for research tasks	7.67 (2.52)	38.9
Support for research from Health Administration		
Positive discrimination measures when applying for funding for research projects	7.56 (2.71)	44.4

**Weaknesses**		
Nursing environment		
Invisibility of research results	8.17 (2.01)	55.6
Difficulty to publish in JCR journals	7.83 (2.77)	55.6
There is a historical weakness of nursing as a science	7.78 (2.32)	55.6
There is no union between research groups and/or research units	7.89 (1.90)	50.0
Few renowned national research groups	7.83 (2.01)	50.0
"Research in nursing care" is usually confused with "evidence-based nursing practice"	6.83 (2.90)	44.4
Difficulties to publish research results in languages other than Spanish	7.72 (2.52)	33.3
There were no lines of research in nursing until recently	7.11 (2.22)	33.3
The scientific quality criteria do not take into account emerging areas in research such as nursing	7.33 (2.20	27.8
Nursing research groups have been created very late in Spain	7.06 (2.18)	22.2
Support for research from Health Administration		
Lack of funding for research projects and for personnel to carry them out	8.33 (1.53)	55.6
It is difficult to apply for funding because nursing applicant's resumes are not competitive enough	7.72 (2.19)	44.4
Academic background		
Lack of undergraduate or graduate research training	7.67 (2.03)	44.4
Little ability to read studies in languages other than Spanish	7.61 (1.94)	33.3
Nursing profession		
Most nurses are women (feminization of the profession) and base of gender inequality	8.17 (1.38)	50.0
To conceive nursing as a teaching-assistance process without considering research as a part of their own	7.78 (1.99)	50.0
Little corporatism among nursing professionals	7.72 (2.19)	50.0
Interest in anything: lack of involvement, dedication, sacrifice and responsibility	7.61 (2.17)	44.4
Nursing invisibility	7.17 (2.18)	27.8
Phobia of theoretical studies	7.22 (1.87)	22.2
**Threats**		
Nursing environment		
Fear of sharing ideas, projects and results	6.33 (2.35)	16.7
Health administration		
Lack of recognition of nursing by hospital management and health administration	8.61 (2.57)	77.8
There is a lack of flexibility at work to devote part of the working day to research	8.39 (2.20)	66.7
Work shifts and care load	8.22 (2.07)	61.1
Registered nurses do not want to work outside the workday to develop research	7.56 (2.06)	38.9

PhD issues		
Shortage of Doctors in Nursing with the ministry autorization to direct a doctoral thesis in nursing	8.22 (2.49)	61.1
Shortage of Doctors in Nursing who can achieve a lecturer/professor position at the Master level	7.22 (2.79)	44.4
There are hardly any Doctors in Nursing to lead other nursing doctoral students	7.89 (1.91)	38.9
Having performed PhD in other disciplines has resulted in that many nurses have stayed in non-nursing research groups and the research results go along those other lines	7.28 (2.24)	38.9
Prior to the implementation of the EHEA, not being able to do PhD programs in nursing (PhD were performed in other disciplines)	6.72 (3.01)	38.9
Academic background		
Lectures in reseach methodology at the undegraduate university level are scarce	7.61 (2.12)	44.4
Critical thinking deficit in the nursing professional group	7.78 (1.70)	33.3
Studies of weak scientific rigor are being carried out, leading to the false belief that nurses are able to investigate	6.39 (2.43)	22.2
Nursing profession		
Lack of knowledge of the usefulness of research among nursing professionals	8.11 (1.57)	55.6
The existence of a glass ceiling for nursing discipline	8.11 (1.78)	44.4
It is not recognized that nursing can investigate; nursing is considered a trade, not a science	8.00 (1.85)	38.9

Abbreviations: SD = standard deviation; PhD = Doctor; EHEA = European Higher Education Area.
